# The Highly Uniform Photoresponsivity from Visible to Near IR Light in Sb_2_Te_3_ Flakes

**DOI:** 10.3390/s21041535

**Published:** 2021-02-23

**Authors:** Shiu-Ming Huang, Jai-Lung Hung, Mitch Chou, Chi-Yang Chen, Fang-Chen Liu, Ruei-San Chen

**Affiliations:** 1 Department of Physics, National Sun Yat-sen University, Kaohsiung 80424, Taiwan; Boos123boos123b@gmail.com; 2 Center of Crystal Research, National Sun Yat-sen University, Kaohsiung 80424, Taiwan; mitch@faculty.nsysu.edu.tw; 3 Department of Materials and Optoelectronic Science, National Sun Yat-sen University, Kaohsiung 80424, Taiwan; 4 Taiwan Consortium of Emergent Crystalline Materials, TCECM, National Sun Yat-sen University, Kaohsiung 80424, Taiwan; 5 Graduate Institute of Applied Science and Technology, National Taiwan University of Science and Technology, Taipei 10607, Taiwan; a0979660804@gmail.com (C.-Y.C.); ls950622@gmail.com (F.-C.L.); rsc@mail.ntust.edu.tw (R.-S.C.)

**Keywords:** Sb_2_Te_3_, broadband photodetector, uniform responsivity

## Abstract

Broadband photosensors have been widely studied in various kinds of materials. Experimental results have revealed strong wavelength-dependent photoresponses in all previous reports. This limits the potential application of broadband photosensors. Therefore, finding a wavelength-insensitive photosensor is imperative in this application. Photocurrent measurements were performed in Sb_2_Te_3_ flakes at various wavelengths ranging from visible to near IR light. The measured photocurrent change was insensitive to wavelengths from 300 to 1000 nm. The observed wavelength response deviation was lower than that in all previous reports. Our results show that the corresponding energies of these photocurrent peaks are consistent with the energy difference of the density of state peaks between conduction and valence bands. This suggests that the observed photocurrent originates from these band structure peak transitions under light illumination. Contrary to the most common explanation that observed broadband photocurrent carrier is mainly from the surface state in low-dimensional materials, our experimental result suggests that bulk state band structure is the main source of the observed photocurrent and dominates the broadband photocurrent.

## 1. Introduction

The interaction of light with matter has been a widely used technique for light sensors and is widely used in our daily life and scientific research. A system with higher photo-interaction efficiency would lead to higher sensitivity. Therefore, there has been increasing interest in finding a material with higher photoresponsivity. The material band structure is a critical factor to finding the efficiency of the light response and light wavelength. As well as the band structure, the light penetration depth is short, so the photon interaction mainly occurs on the material surface. To enhance the photon interaction, a system with higher surface area is preferred [1,2,3,4,5,6]. In addition to the higher surface ratio, earlier studies have demonstrated that carrier mobility is one of the most critical factors for determining the light responsivity [7,8]. With these considerations in mind, nanostructures and low-dimensional systems with high mobility have been widely investigated [9,10,11,12,13,14,15,16]. Experimental results have revealed that most materials only show high photoresponsivity at particular wavelengths due to their specific band structure, and this limits their potential application. A broadband response along with a high photoresponsivity is important for the potential application across a wide range of wavelengths.

A system with a linear energy-momentum band structure, such as graphene, graphene-based heterostructures, and topological materials, could satisfy these requirements and provide a high photoresponsivity over a broadband wavelength range. Many studies have demonstrated high photoresponsivity over a wide range of wavelengths in these systems. The graphene/transition metal dichalcogenide system shows an extremely fast photoresponse [17]. InGaAs shows a photoresponse for light wavelengths ranging from 400 to 16,000 nm [18]. However, previous reports have revealed that the photoresponses show a strong wavelength-dependent deviation in these systems [10,15,19,20,21,22,23,24,25,26,27,28,29,30,31,32,33,34,35,36,37,38,39,40,41,42,43,44,45,46,47,48,49]. This inhomogeneous response would limit the potential applications of these materials as broadband photosensors. The search for a system with uniform light responsivity over a wide range of wavelengths is one of the on-going topics in the field of light sensor applications. The light penetration into the material and carriers from the bulk state of the topological material might also contribute to the measured photocurrent. This might be one of the reasons behind the observed strong wavelength dependence in the previous reports [50]. To solve this problem, as well as the surface state band structure, one might take the bulk state band structure into account. We checked the bulk state band structure of BixSb(2−x)TeySe(3−y) topological materials and found that Sb_2_Te_3_ might be a good candidate to providing uniformity of the photocurrent over a wide range of wavelengths. 

In this work, we study the photoresponse of Sb_2_Te_3_ flakes. Our results show that the photocurrent is linear with the light power and the applied electric voltage, and that the photoresponsivity is proportional to the conductance of the flake. According to previous reports, the photoresponsivity is stable for light wavelengths ranging from visible to near-infrared light, and the normalized root means that the square photoresponsivity deviation is about 6%, which is much lower than reported results in low-dimensional systems. The results show that the corresponding energy of the measured photocurrent peaks and dips at different light wavelengths are consistent with the energy difference of the carrier density of state peaks and dips between the conduction and valence band of Sb_2_Te_3_ bulk state. This result supports that the observed photoresponsivity is dominated by the carriers of the bulk state and not from the topological surface state. The low-deviated band structure of bulk state leads to the behavior of the observed uniform broadband light responsivity.

## 2. Materials and Methods

High purity elements Sb (99.995%) and Te (99.995%) were mixed according to the stoichiometric ratio in a glove box with low oxygen and water to avoid oxidation. The mixed raw materials were put into a vacuum quartz glass tube (pressure ~ 10^−3^ torr) and sealed inside the quartz glass tube. After that, the materials were melted at 750 °C for 20 h, and then slowly cooled down to 625 °C in 5 h. It took another 60 h to cool the materials down to 565 °C at the cooling rate 1 °C/h. The melting point of Sb_2_Te_3_ is 617 °C, so this slow cooling rate ensured that Sb_2_Te_3_ could be slowly crystalized. Then, it took 10 h to cool the materials down to room temperature. The crystallized Sb_2_Te_3_ was further purified and crystallized by a homemade resistance-heated floating zone furnace (RHFZ). The Sb_2_Te_3_ was locally heated up to 617 °C and the Sb_2_Te_3_ formed an interface of solid phase and liquid phase. At the same time, we slowly moved the quartz glass tube and changed the position of the solid/liquid interface. During this process, the impurities were moved with the liquid phase and were excluded from the recrystallized Sb_2_Te_3_. After this recrystallization process, single crystals of Sb_2_Te_3_were naturally cooled down to room temperature. The as-grown crystals were cleaved along the basal plane, producing a silvery shining mirror-like surface, and then prepared for further experiments. The Raman spectrum and electron probe microanalyzer (EPMA) spectrum support that the produced crystal was Sb_2_Te_3_ [51]. Details of Raman spectrum and EPMA spectrum are given in Appendix A. Sb_2_Te_3_ flakes were obtained by exfoliating bulk crystals using dicing tape and were then dispersed on the insulating SiO_2_ (300 nm)/n-Si templates with prepatterned Ti/Au circuits. Two platinum (Pt) metal contacts were subsequently deposited on the selected Sb_2_Te_3_ flakes using focused-ion beam (FIB) technique. The Chemical Abstracts Service (CAS) numbers of all the chemical and substrates are given in Appendix B. Details of the x-ray diffraction (XRD), Raman, EPMA, SEM, and electrical measurement system are given in Appendix C.

## 3. Results and Discussion

**Figure 1:**
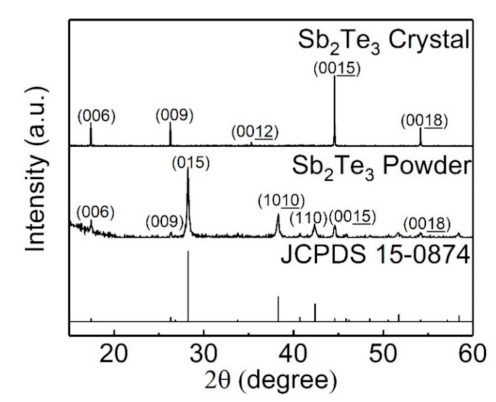
The X-ray diffraction (XRD) pattern of the produced Sb_2_Te_3_. The sharp peaks indicate that the Sb_2_Te_3_ is highly single crystalline. The Sb_2_Te_3_ powder was ground from the Sb_2_Te_3_ crystal. The XRD pattern of the Sb_2_Te_3_ powder is consistent with the JCPDS card.

Figure 1 shows the XRD pattern of the produced Sb_2_Te_3_. The Sb_2_Te_3_ crystal showed sharp peaks. It revealed the Laue diffraction peaks at the c-axis. The crystal was grown on the [001] direction. The full width at half maximum (FWHM) was between 0.055 and 0.06 deg. The average crystallite size at the c-axis was about 124 nm and the strain was −0.018%. These results indicate that the Sb_2_Te_3_ was highly single crystallized. The Sb_2_Te_3_ powder was ground from the Sb_2_Te_3_ crystal. The XRD pattern of the Sb_2_Te_3_ powder was consistent with the Joint Committee on Powder Diffraction Standards (JCPDS) card. 

**Figure 2:**
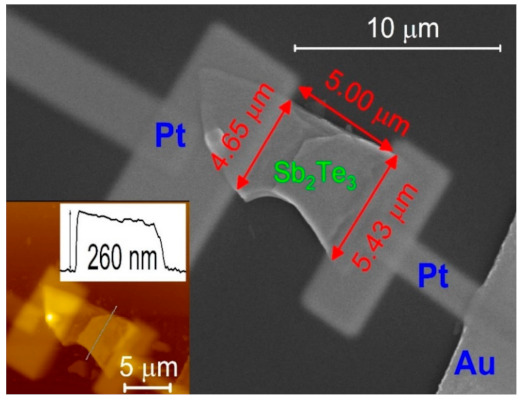
The SEM picture of the Sb_2_Te_3_ flake for the photocurrent experiment. Two Pt contacts were deposited on the Sb_2_Te_3_ flake. The left-bottom inset shows an Atomic Force Microscope (AFM) picture of the Sb_2_Te_3_ flake and the AFM thickness profile of the flake. The thickness is about 260 nm.

Figure 2 shows the SEM image of a Sb_2_Te_3_ flake. It is 5 µm long and ∼5 µm wide. The thickness of a flake is determined by the atomic force microscopy; here, the flake was 260 nm thick (shown in the left-bottom inset of Figure 2).

**Figure 3:**
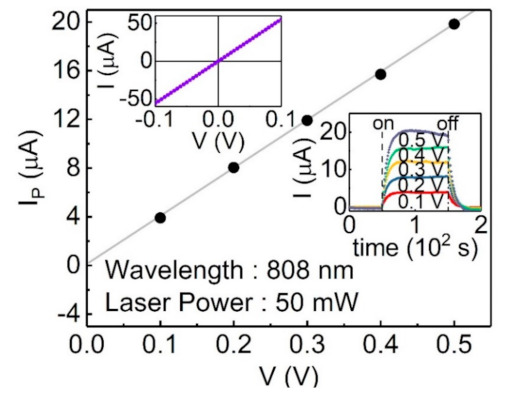
The left-top inset shows the linear current–voltage relationship, which indicates the ohmic contact between the Sb_2_Te_3_ flake and Pt electrodes. The right-bottom inset shows the measured photocurrent at different applied voltages. The main figure shows the extracted photocurrent as a function of applied voltage. The photocurrent varies linearly with the applied voltage.

The left-top inset of Figure 3 shows the linear current–voltage relationship of the flake; the conductivity is about 20.4 S/cm. The right-bottom inset of Figure 3 shows the on–off photocurrent response at different applied electric voltages under 808 nm light wavelength and 50 mW laser power. In this work, we define the photocurrent as the measured current difference between the condition with and without light illumination. The main image from Figure 3 shows that the extracted photocurrent, $I_{P}$, is linear with the applied electric voltage. These results indicate the ohmic contacts between the Sb_2_Te_3_ flake and Pt electrodes, and the metallic behavior of the Sb_2_Te_3_ flake.

**Figure 4:**
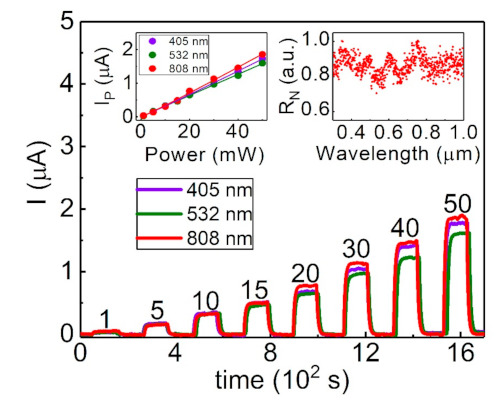
The photocurrent as a function of the light power at three wavelengths. The left-top inset shows that the photocurrent varies linearly with the light power at three wavelengths. The right-top inset shows that the responsivity is insensitive to wavelengths from 300 to 1000 nm.

Figure 4 shows the measured current of our Sb_2_Te_3_ flake under light illumination with light power ranging from 1 to 50 mW, which corresponds to the power intensity of 40 to 2000 Wm^−2^. The photocurrent increases with increasing light power and shows a similar power-dependent photocurrent under three different light wavelengths. The left-top inset of Figure 4 reveals the extracted photocurrent as a function of the light power at three different light wavelengths. It clearly reveals that the photocurrent varies linearly with the light power for three light wavelengths. The higher light intensity means higher photon interaction. The increasing amount of photon interaction will induce more excited carriers, leading to a higher photocurrent. The light intensity is not over the limit of the excited carrier number, and the measured photocurrent will be proportional to the increasing light intensity, resulting in a positive slope for the three different light wavelengths.

To further identify this characteristic in the three different wavelengths, the photocurrent is expressed as the simple power-law relation $I_{P}\;=\;\alpha P^{\beta }$, where the *α* is a constant for the wavelength of the illuminating light, *P* is the illuminated light power on the Sb_2_Te_3_ flake, and *β* is a constant related to the transport mechanism and the intrinsic optic characteristics. Table 1 lists *α* and *β* for the three different wavelengths. The result reveals that *β* is 1.03 ± 0.02, which indicates that the behavior of the laser power-dependent photocurrent of the system remains the same at different light wavelengths. The complex carrier scattering process might lead to the non-integer *β*. Our experiment shows *β*∼1, which indicates that the carrier transport process is insensitive to the extrinsic light power and interaction between excited carriers. This characteristic is more flexible and stable for a wide range of potential applications. The broadband light response has been observed in many low-dimensional systems [10,15,19,20,21,22,23,24,25,26,27,28,29,30,31,32,33,34,35,36,37,38,39,40,41,42,43,44,45,46,47,48,49]. These reports show that the observed photocurrent is strongly dependent on light wavelength, which would limit the application potential of the broadband light sensor. Advancing the findings of previous reports, our experimental results reveal that the photocurrent is weakly wavelength dependent [10,15,19,20,21,22,23,24,25,26,27,28,29,30,31,32,33,34,35,36,37,38,39,40,41,42,43,44,45,46,47,48,49]. The *α* roughly deviates by 30% at the three different wavelengths, which is much smaller than the reported values. The light power and the measured photocurrent depended upon the effective geometric area of the material. In order to quantitatively determine the performance of the Sb_2_Te_3_ flake under illumination, the responsivity, *R*, is calculated through the following equation:

(1) $$R\;=\frac{I_{P}}{PS},$$

where $I_{P}$, *P,* and *S* are the photocurrent, the light intensity, and the effective area, respectively. The obtained *R* values are 38, 31, and 43 AW^−1^ for the wavelengths of 405, 532, and 808 nm at a constant applied voltage of 0.1 V. The deviation ratio is about 34%. 

To further examine the photocurrent response characteristic at wide wavelength, a photocurrent spectrum measurement was performed. As shown in the top-right inset of Figure 4, the *R* reveals weak wavelength dependence over a range of wavelengths. The normalized root means square is used to identify the deviation level. The normalized root means square is 0.06, which indicates a 6% deviation for the light wavelength ranging from 300 to 1000 nm. This behavior is more stable than previously reported results in low-dimensional systems. Along with the responsivity, the responsivity stability is a critical factor for the application of broadband photosensors. Table 2 lists the reported broadband light responsivity of various kinds of materials. It shows that most of these materials reveal a large light response deviation on the broadband light wavelength. The photo deviation of the Sb_2_Te_3_ flake is a few orders smaller than the reported values of various materials.

**Figure 5:**
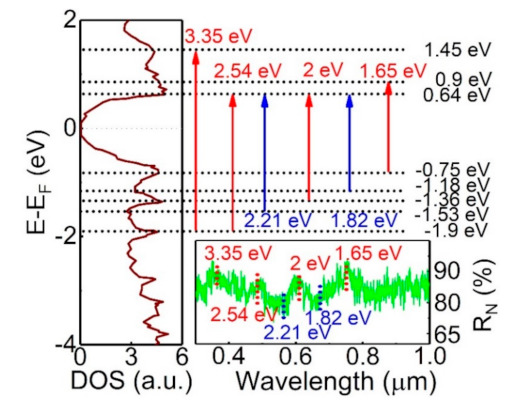
The left side shows the band structure of the Sb_2_Te_3_ bulk state. The right side shows the photocurrent as a function of light wavelength. The corresponding energies of these photocurrent peaks at different light wavelengths are consistent with the energy difference of band structure peaks between conduction and valence bands.

The left side of Figure 5 reveals the density of state of the Sb_2_Te_3_ bulk state, and it reveals several peaks at valence and conduction bands. The right side of Figure 5 shows the measured photoresponsivity as a function of light wavelength, and it exhibits several photocurrent peaks at different wavelengths. Our analysis shows that the corresponding energies of these measured photoresponsivity peaks and dips at different light wavelengths are consistent with the energy difference of Density of states (DOS) peaks between conduction and valence bands. This suggests that the observed photocurrent originates from these band structure peak transitions under light illumination. Contrary to most previous explanations that the observed broadband photocurrent carrier is mainly from the surface state in topological materials, our experimental result suggests that bulk state carriers are the main source of the observed photocurrent and dominate the broadband photocurrent.

**Figure 6:**
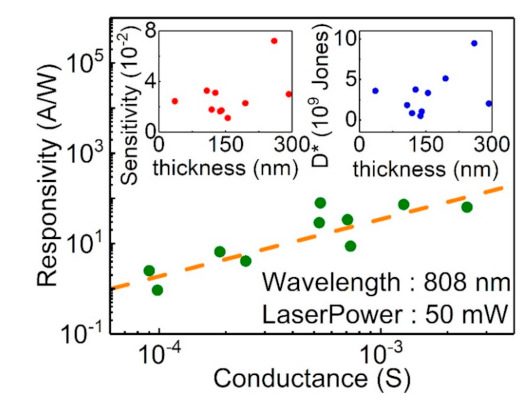
The responsivity is proportional to the conductance of the flake. The top-left inset shows that the responsivity is insensitive to the flake thickness. The top-right inset shows that the detectivity is insensitive to the flake thickness.

The photocurrent is directly related to the effective carrier mobility, which is determined by both the carriers from the bulk state and surface state. Figure 6 shows the photocurrent responsivity as a function of the conductance of the Sb_2_Te_3_ flakes. It shows that the photocurrent responsivity is proportional to the conductance of the Sb_2_Te_3_ flakes. This implies that effective conductance is a critical factor to determining the photocurrent responsivity. Detectivity, which is an important figure of merit in evaluating the ability of a photodetector to detect a weak signal, is another important index used to characterize the performance of photodetectors. The specific detectivity ($D^{*}$) is calculated through the following equation:

(2) $$D^{*}=\frac{RS^{1/2}}{{\left(2qI_{d}\right)}^{1/2}},$$

where *R*, *S*, *q*, and $I_{d}$ are the responsivity, effective area of light illumination, electronic charge, and dark current, respectively. By using the experimental data, the obtained detectivity is about $2\;\times 10^{9}$ Jones. As shown in the top-right inset of Figure 6, the detectivity is almost insensitive to the flake thickness. The top-left inset of Figure 6 shows that the sensitivity is about 0.02. Our experimental results show that the detectivity and the sensitivity is insensitive to the flake thickness. However, it should be noted that the light penetration length is about 20 nm, which is much shorter than the thickness of our Sb_2_Te_3_ flakes. This might be the reason behind the observed thickness-independent detectivity and sensitivity.

## 4. Conclusions

The photocurrent measurement was performed on Sb_2_Te_3_ flakes at various wavelengths ranging from visible to the near IR region. The photocurrent was observed to vary linearly with the applied electric voltage, light power, and conductance of the flakes. Advancing the findings of previous reports that the photoresponse reveals strong wavelength dependence, our measured photocurrent was insensitive to wavelengths from 300 to 1000 nm. The wavelength response deviation was lower than that in all previously reported photoresponse materials and it has the potential to play a role in broadband photosensors. Our analysis showed that the corresponding energies of these photocurrent peaks were consistent with the energy difference of the density of state peaks between conduction and valence bands. This further suggests that the observed photocurrent originated from these band structure peak transitions under light illumination. Contrary to the most common explanation that the observed broadband photocurrent carrier is mainly from the surface state in low-dimensional materials, our experimental result suggests that the bulk state band structure is the main source of the observed photocurrent and dominates the broadband photocurrent. As well as the linear energy-momentum dispersion, one has to take the bulk state band structure into account for the broadband photo-detection material.

## Figures and Tables

**Table 1:** List of the fitting parameters of the laser power-dependent photocurrent for three light wavelengths.

Laser Wavelength	405 (nm)	532 (nm)	808 (nm)
$\alpha \;\left(A/W\right)$	38	31	43
β	1.03	0.99	1.05

**Table 2:** List of the wavelength range and the deviation ratio of photocurrent for reported broadband light materials.

Material	Wavelength (nm)	Deviation Ratio (%)	Reference
Sb_2_Te_3_	300∼1000	34	This work
Sb_2_Te_3_/Si/Sb_2_Te_3_	365∼940	98.17	[19]
Si	350∼1050	109.41	[20]
InSe	400∼800	180	[21]
InSe	370∼980	266	[22]
In_2_Se_3_	250∼700	252	[23]
In_2_Se_3_	400∼900	104	[24]
AsP	2500∼8000	121	[25]
BP	300∼680	2449	[26]
BP	405∼940	156	[27]
MoSe_2_	400∼600	87	[28]
WSe_2_	500∼800	217	[29]
WSe_2_	390∼880	70	[30]
WS_2_	450∼650	235	[31]
MoS_2_	400∼690	542	[32]
MoS_2_	455∼850	178	[15]
PtSe_2_	2500∼10,000	373	[33]
SnS_2_	300∼700	130	[34]
SnSe_2_	500∼700	286	[35]
Graphene	600∼9600	7334	[36]
Graphene	1100∼1650	1443	[37]
GaSe	220∼650	165	[38]
GaSe	400∼800	368	[39]
GaS	245∼610	278	[10]
CoSe	450∼4100	143	[40]
CoSe	450∼950	132	[40]
PbS	400∼2000	186	[41]
InGaAs	400∼16,000	397	[18]
InGaAs	400∼1000	220	[18]
Sb_2_Se_3_	300∼1100	267	[42]
Sb_2_Se_3_	300∼1100	226	[43]
In_2_Te_3_	350∼1090	129	[44]
In_2_Te_3_	350∼1000	403	[45]
SnTe	405∼2003	120	[46]
SnTe	405∼808	91	[46]
SnTe	254∼650	399	[47]
Cd_3_As_2_	532∼10,600	926	[48]
Cd_3_As_2_	532∼940	140	[48]
EuBiTe_3_	370∼1550	112	[49]
EuBiTe_3_	370∼1064	110	[49]

## Data Availability

Not applicable.

## Appendix A

The details of Raman spectrum and EPMA: The Raman spectrum shows five peaks, E^1^_g_: 44.40 cm^−1^, E^2^_g_: 113.96 cm^−1^, A_1_(Te): 126.33 cm^−1^, E^H^(Te): 141.72 cm^−1^, and A^1^_1g_: 165.46 cm^−1^. That is consistent with Sb_2_Te_3_ oscillation frequencies from previous reports [52,53]. EPMA shows that the ratio of Sb to Te at different portions of the single crystal ranges from Sb:Te = 1.95:3 to 2.00:3.

## Appendix B

The CAS numbers of all the chemical and substrates: Antimony (Sb) CAS NO.7440-36-0; Tellurium (Te) CAS NO.13494-80-9; Silicon (Si) CAS NO.7440-21-3; Silicon dioxide (SiO_2_) CAS NO.7631-86-9; Platinum (Pt) CAS NO.7440-06-4; Gold (Au) CAS NO.7440-57-5; Titanium (Ti) CAS NO.7440-32-6.

## Appendix C

X-Ray Diffraction (XRD):

Equipment Model: Siemens D5000 (for powder diffraction measurement); Target: Copper (Cu), Wavelength ($\lambda_{Ka}$) = 0.10540598 nm; Output Power: Voltage = 40 kV, Current = 30 mA; Step Size: 0.05°; Step Time: 1.5 s; Scan Range: 15°~60°.

Equipment Model: Bruker D8 (For Crystal diffraction Measurement); Target: Copper (Cu), Wavelength ($\lambda_{Ka}$) = 0.10540598 nm; Output Power: Voltage = 40 kV, Current = 40 mA; Step Size: 0.01°; Step Time: 0.5 s; Scan Range: 15°~60°.

Raman:

Equipment Model: Micro-Raman Spectroscopy HR800; Input Laser: He-Cd laser; Wavelength: 633 nm; Scan Range: 25~225 cm^−1^.

Electron Probe Microanalyzer (EPMA):

Equipment Model: JEOL JXA-8530F Field Emission Electron Probe Microanalyzer; Accelerating Voltage: 10 kV; Magnification: 5000×.

Scanning Electron Microscope (SEM):

Equipment Model: FEI Quanta 3D FEG; Accelerating Voltage: 10 kV; Magnification: 3500×.

Electrical Measurement System:

Equipment Model: Keithley 4200-SCS; Source Type: Voltage; 210; Voltage Range: −0.1~0.1 V and0.005 V Step for Current–Voltage Measurement; Voltage: 0.1 V, 0.2 V, 0.3 V, 0.4 V, 0.5 V and 1 s Step for Current–Time Measurement.
